# Hartshorne—Essentials of Practice

**Published:** 1874-11

**Authors:** 


Essentials of the Principles and Practice of Medicine. A hand-book for Students and Practitioners. By Henry Hartshorne. A.M. M.D., Professor of Hygiene in the University of Pennsylvania, etc. Fourth Edition, thoroughly Revised; with 100 Illustrations. Philadelphia: Henry C. Lea; 1874; 12mo; cloth; pp. 548. Price $2.63. Sold in Atlanta by M. Lynch & Co.

The marked success which this hand-book has achieved in the short space of seven years is the best evidence that it satisfactorily supplies a want generally felt by the medical profession of America. This very gratifying evidence of appreciation of his labors by his brethren has stimulated the author to make a thorough revision in the present edition, introducing cold baths, in fevers, pneumatic aspiration, transfusion of blood, recto-abdominal exploration, of Simon, and other novelties of practice, together with one hundred illustrations, which have not been presented in previous editions.

It is, what its title imports, an admirable hand-book of the *essentials* of the principles and practice of medicine, and will be found a fitting companion of the popular hand-book of surgery of Dr. Druitt, of London.

